# Variation in outcomes after metabolic bariatric surgery: multilevel analysis to assess the contribution of patient, surgeon, and hospital factors

**DOI:** 10.1093/bjs/znaf186

**Published:** 2025-10-03

**Authors:** Floris F E Bruinsma, Simon W Nienhuijs, Ronald S L Liem, Jan Willem M Greve, Perla J Marang-van de Mheen, the Dutch Audit for Treatment of Obesity Research Group, G J D van Acker, J Apers, S C Bruin, F F E Bruinsma, S M M de Castro, S L Damen, I F Faneyte, J W M Greve, G van ‘t Hof, F H W Jonker, R A Klaassen, E A G L Lagae, B S Langenhoff, R S L Liem, A A P M Luijten, S W Nienhuijs, R M Smeenk, S J M Smeets, W Vening, M Takkenberg, E de Witte

**Affiliations:** Department of Surgery, Maastricht University Medical Centre, NUTRIM School for Nutrition and Translational Research in Metabolism, Maastricht, The Netherlands; Scientific Bureau, Dutch Institute for Clinical Auditing, Leiden, The Netherlands; Department of Surgery, Catharina Hospital, Eindhoven, The Netherlands; Department of Surgery, Groene Hart Hospital, Gouda, The Netherlands; Department of Surgery, Nederlandse Obesitas Kliniek, The Hague and Gouda, The Netherlands; Department of Surgery, Maastricht University Medical Centre, NUTRIM School for Nutrition and Translational Research in Metabolism, Maastricht, The Netherlands; Weight Doctors Nederland, Quole, Waalre, The Netherlands; Safety & Security Science and Centre for Safety in Healthcare, Delft University of Technology, Delft, The Netherlands

## Abstract

**Background:**

Metabolic bariatric surgery (MBS) quality registries monitor various outcomes, enabling the assessment of hospital performance in comparison with national benchmarks. However, if there is considerable between-surgeon outcome variation, surgeon-level feedback may be better suited. The aim of this study was to assess the extent to which patient-, surgeon-, and hospital-level factors contribute to the variation in outcomes after MBS.

**Methods:**

All primary procedures registered in the Dutch MBS quality registry between 1 January 2020 and 31 December 2023 were included. Outcomes included severe postoperative complications, reoperation, prolonged length of stay (LOS), readmission, textbook outcome, and achieving ≥25% total weight loss within 1 year. Multilevel logistic regression models were built for each outcome, including all available patient characteristics, operating surgeon, and hospital, to determine the variance explained by patient-, surgeon-, and hospital-level factors.

**Results:**

In total, 30 610 patients were included, operated on by 144 surgeons in 19 hospitals. Hospital-level factors contributed most to the explained variance for all outcomes, ranging from 59.6% for reoperation to 90.3% for prolonged LOS. Surgeon-level factors explained less variance, ranging from 3.2% for prolonged LOS to 28.2% for reoperation. Patient characteristics explained the least, ranging from 4.4% for textbook outcome to 13.1% for severe postoperative complications.

**Conclusion:**

Variation in outcomes is mostly explained by hospital factors, rather than surgeon factors, supporting hospital-based performance feedback. The results suggest that the pre- and postoperative trajectory and perioperative care may affect MBS outcomes more than patient characteristics or surgical team performance.

## Teaser

Variation in outcomes after metabolic bariatric surgery (MBS) is influenced by multiple factors, including patient characteristics, surgeon expertise, and hospital practices. However, the relative impact of each factor has remained unclear. In an analysis of six key MBS outcomes among 30 610 patients, hospital-level factors accounted for the largest share of the explained variance across all outcomes, followed by surgeon-level and then patient-level factors.

## Introduction

Outcomes after metabolic bariatric surgery (MBS) are influenced by a complex interplay of patient characteristics, surgical team performance, and institutional practices such as local protocols and care processes. Previous research has primarily focused on patient characteristics, identifying numerous patient factors that are linked to patient outcomes. For instance, diabetes is associated with lower weight loss^1,2^, while older age, male sex, and a history of pulmonary and cardiovascular disease, among others, increase the risk of postoperative complications^3,4^. Additionally, patients with gastro-oesophageal reflux disease or diabetes benefit more from gastric bypass than sleeve gastrectomy^5–8^. These insights have enhanced patient-tailored care, enabling more accurate outcome predictions, improved preparedness, and better preoperative counselling. Research on patient characteristics has yielded these valuable insights, but greater emphasis may be needed on other factors, as patient outcomes are also influenced by the quality of delivered care.

The experience and skill of the surgeon and surgical team are associated with the chance of postoperative complications, both in general and in MBS surgery^9–16^. Moreover, the surgeon plays a role in deciding which type of surgery is performed^17^, which can be viewed as a quality-of-care indicator to select the best type of surgery for a particular patient, contributing to outcome differences. Next to these surgery-related factors, outcome variation between hospitals is regularly observed and often persists after case-mix adjustment, indicating systematic differences in performance between hospitals that are unrelated to patient factors^18–22^.

While it is well established that all three elements—patient, surgeon together with surgical team, and hospital—contribute to postoperative outcomes, the relative contribution of each to the overall variation in MBS outcomes remains unclear. This is relevant because, currently, feedback on performance is mainly given to hospitals, whereas if most of the variation is due to systematic differences in performance between surgeons, it would be better delivered to surgeons in order to improve care. Therefore, the aim of this study was to assess the extent to which patient-, surgeon-, and hospital-level factors contribute to the variation in outcomes after MBS, providing critical insights into where targeted efforts may have the greatest impact with regard to optimizing MBS care.

## Methods

### Setting and patient selection

Patients were selected from the Dutch Audit for Treatment of Obesity (DATO)^23^. This mandatory MBS quality registry started collecting patient data in 2015^24^. Collected data include information on demographics, procedures, complications, and weight loss outcomes, with the surgeon performing the surgery also registered. All surgeons are assigned a hospital-specific identifier by their hospital for registering procedures in DATO, starting from one for surgeons, with the higher numbers (for example 99) reserved for residents and fellows. The identity linked to each identifier is known only within the surgeon’s hospital, allowing outcomes and patient mix to be compared with those of the surgeon’s immediate colleagues, while retaining anonymization for research purposes. All patients receiving primary MBS between 1 January 2020 and 31 December 2023 were included. Patients were excluded if the surgeon or hospital was not registered or if baseline patient characteristics were missing (see below).

The study was approved by the scientific committee of DATO and the local science office of the Dutch Institute for Clinical Auditing and was performed in accordance with ethical standards as stated in the Declaration of Helsinki and its later amendments^25^. Following Dutch law, no additional informed consent was required, as DATO is an opt-out registry that uses strictly pseudonymized data.

### Patient characteristics and outcomes

The following baseline patient characteristics were included: presence of diabetes mellitus, hypertension, dyslipidaemia, obstructive sleep apnoea syndrome, gastro-oesophageal reflux disease, musculoskeletal pain, age (categorized as <35, 35–64, and ≥65 years old), sex, BMI (categorized as <40 and ≥40 kg/m^2^), ASA grade (categorized as <III and ≥III), and Charlson co-morbidity index^26^ (a score to predict overall survival based on characteristics such as cardiovascular disease, pulmonary disease, and other systemic diseases significantly affecting overall survival; categorized as 0, 1, and ≥2).

The investigated outcomes were severe postoperative complications (Clavien–Dindo grade ≥3)^27,28^, reoperation within 30 days, prolonged (≥3 days) length of stay (LOS), readmission within 30 days, textbook outcome (that is a composite outcome measure indicating no prolonged LOS and no readmissions or complications within the first 30 days)^29^, and achieving ≥25% total weight loss (TWL) 1 year after surgery.

### Statistical analysis

First, stepwise forward logistic regression analysis was used, including all available patient covariates, to determine the relevant patient characteristics influencing the outcome of interest and achieving the best model fit. The model fit was determined by evaluating the Akaike Information Criterion (AIC), with a lower AIC indicating a better model fit.

Second, a multilevel null model was created for each outcome, including only the surgeon variable, to determine the between-surgeon variance. The surgeon variable reflects those aspects of care shared by patients operated on by the same surgeon. The relevant patient covariates were then added to the multilevel model and the absolute change in between-surgeon variance was documented as the within-surgeon variance (that is the variance explained by adding the patient-level characteristics).

Third, the hospital level was added to the multilevel model containing surgeon and patient characteristics and, from this model, the final between-surgeon and between-hospital variances were determined. The hospital variable reflects those aspects of care shared by all patients operated on in the same hospital, such as preoperative and postoperative care, and therefore could also reflect care delivered at other locations affiliated with the hospital (registered in DATO as belonging to that hospital). The total variance explained by the multilevel model was computed as the sum of the within-surgeon variance, between-surgeon variance, and between-hospital variance, so that the proportions of the variance contributed by patient-, surgeon-, and hospital-level factors could be calculated.

Finally, the intraclass correlation coefficient (ICC) was determined for the hospital and surgeon levels to put the explained variance into the context of the total variance (including variance not explained by the included factors). For dichotomous outcomes, the ICC is calculated using π²/3 for the variance at the patient level^30^. Stacked bar charts were created for all outcomes to visualize the proportion of explained variance assigned to each level.

### Sensitivity analyses

A supervised surgical resident may be the primary operating surgeon. Often, these residents get their own surgeon identification code assigned by the hospital when registering procedures in DATO, using the higher numbers, for example 90–99. To investigate the impact of residents performing surgery on the variance explained by the surgeon level, two sensitivity analyses were performed. In the first sensitivity analysis, all surgeon identifiers >15 were excluded, as it seemed unlikely that these higher numbers represented bariatric surgeons, given the known number of bariatric surgeons employed by hospitals. In the second sensitivity analysis, all surgeon identifiers with <50 primary procedures in the study interval were excluded, as all Dutch bariatric surgeons are expected to have performed many more in the study interval, so that these identifiers likely reflected guest surgeons, fellows, or residents.

## Results

In total, 39 517 patients were identified, of whom 30 610 remained eligible for analysis after excluding those with incomplete baseline characteristics or missing surgeon identifiers. Patients were operated on by 144 different surgeons, with surgeries conducted in 19 hospitals. The median number of surgeries performed was 152 (interquartile range (i.q.r.) 5–372) per surgeon and 1713 (i.q.r. 752–2108) per hospital.

**Fig. 1:**
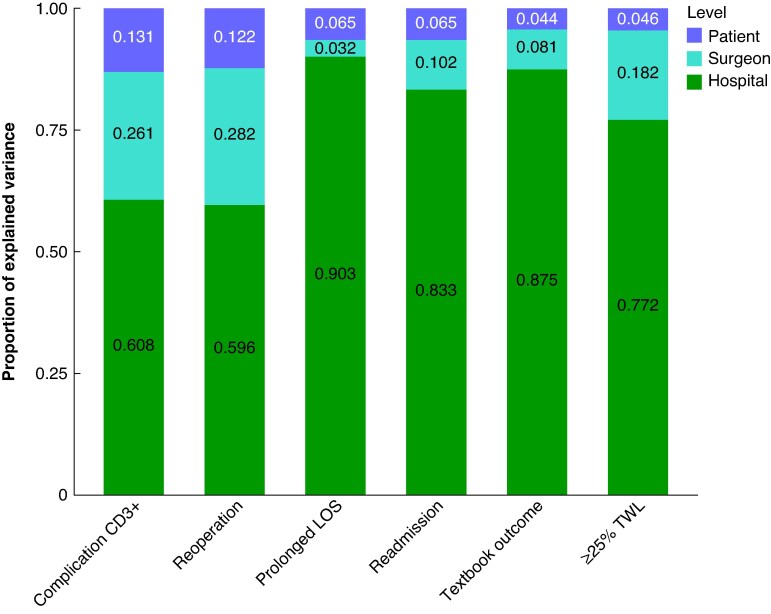
Distribution of the explained variance over the different levels Prolonged LOS is an LOS ≥3 days. Textbook outcome is a composite outcome measure indicating no prolonged LOS and no readmissions or complications within the first 30 days. CD3+, Clavien–Dindo grade ≥3; LOS, length of stay; TWL, total weight loss.

The relative contribution of patient-, surgeon-, and hospital-level factors to the explained variance in outcomes is displayed in *Fig. 1*. Patient characteristics contributed minimally to the explained variance for all outcomes, ranging from 4.4% for textbook outcome to 13.1% for severe postoperative complications. The surgeon level contributed more to the explained variance for most outcomes, ranging from 3.2% for prolonged LOS to 28.2% for reoperation. Only for prolonged LOS was the surgeon-level contribution smaller than the contribution of patient characteristics (3.2% *versus* 6.5% respectively). The hospital level contributed most to the explained variance for all outcomes, ranging from 59.6% for reoperation to 90.3% for prolonged LOS.

Examining the proportion of total variance explained, the surgeon-level ICC was low for all outcomes, indicating that surgeon-level factors explained only a small proportion of the total variance in outcomes; it was highest for reoperation (1.5%) (*Table 1*). The hospital level explained small to moderate parts of the total variance, with the ICC being highest for prolonged LOS (15.2%).

**Table 1:** ICCs for the different outcomes at the surgeon level and at the hospital level

	ICCs	
	Surgeon	Hospital
Complication CD3+	1.2	2.7
Reoperation	1.5	3.2
Prolonged LOS	0.6	15.2
Readmission	0.9	6.7
Textbook outcome	0.5	5.2
≥25% TWL	1.1	4.5

Values are %. Prolonged LOS is an LOS ≥3 days. Textbook outcome is a composite outcome measure indicating no prolonged LOS and no readmissions or complications within the first 30 days. ICCs, intraclass correlation coefficients; CD3+, Clavien–Dindo grade ≥3; LOS, length of stay; TWL, total weight loss.

### Sensitivity analyses

After excluding cases where the surgeon identifier was >15, 30 031 patients operated on by 117 surgeons were included in the first sensitivity analysis (the median number of surgeries performed was 208 (i.q.r. 49–409) per surgeon). There were only slight changes in the proportion of explained variance attributable to each level, with the hospital level still explaining the greatest part of the variance for all outcomes (*Table S1*). After excluding surgeons who performed <50 procedures in the specified interval, 30 235 patients operated on by 89 surgeons were included in the second sensitivity analysis, also with minimal changes in the proportion of variance ascribed to each level (*Table S1*) (the median number of surgeries performed was 323 (i.q.r. 165–447) per surgeon).

## Discussion

The present study showed that the largest part of the explained variance is contributed by hospital-level factors, with surgeon-level factors contributing a considerably smaller part. Patient characteristics contributed minimally to the explained variance in outcomes. These findings suggest that, to improve MBS care further, caregivers should first focus on improving aspects of care shared by all patients in the same hospital, such as the preoperative trajectory and perioperative care. Providing performance feedback thus seems best directed to hospitals. However, adding surgeon-level feedback can be considered for outcomes like reoperations and severe complications, as surgeon-level factors contributed noticeably to the explained variance for these outcomes.

In contemporary literature, significant attention has been devoted to identifying factors associated with outcomes after MBS. Research has primarily focused on patient characteristics, leading to the identification of several risk factors for adverse outcomes^1–4^. However, the relative contribution of patient characteristics *versus* surgeon- and hospital-level characteristics in MBS has remained underexposed. The present study is the first to provide insight into this matter. The limited impact of patient characteristics also reinforces that hospital performance feedback regarding MBS outcomes within the Netherlands does not require adjustment for differences in case mix^23^.

In other surgical disciplines, little attention has also been paid to examining the relative impact of hospital- or surgeon-level factors compared with patient factors on outcomes^9–15^. To the authors’ knowledge, only one study has previously investigated the influence of the surgeon compared with the influence of patient characteristics^31^. In that study, surgeon-specific factors were found to have a greater impact on partial nephrectomy outcomes, such as positive surgical margins and complications, than patient-related factors, which aligns with the findings of the present study. However, that study was not able to assess differences in hospital performance, so the relative contributions of surgeon-specific factors and patient-related factors compared with the relative contribution of overarching hospital-related factors remained unclear. In another study on colectomies, differences in complication rates between hospitals and surgeons were examined, revealing a wider variation in complication rates among surgeons than between hospitals; this finding led Xu *et al*.^32^ to conclude that there is greater improvement potential at the surgeon level. However, hospital rates are based on the results of multiple surgeons combined and Xu *et al*.^32^ did not investigate the relative impact of hospitals *versus* surgeons. As colectomies have a higher a priori risk of complications and are generally performed at lower volumes per surgeon^33^, it is plausible that differences in surgeon experience and technique (for example laparoscopy *versus* laparotomy) play a more prominent role. In the Netherlands, clear guidelines mandate that each MBS hospital must have a minimum of two dedicated bariatric surgeons and perform ≥200 primary MBS procedures annually^34^, which ensures a minimum surgical standard. This may explain why the present study showed that differences at the hospital level contributed more than differences at the surgeon level. Nevertheless, the guidelines are also intended to ensure that departments are adequately equipped to provide optimal patient support throughout the preoperative and postoperative phases. However, this has not resulted in fully standardized protocols across institutions and variations in outcomes persist, even in a well-developed and organized healthcare system such as that of the Netherlands.

The low median number of surgeries performed per surgeon in the primary analysis (IQR starting at 5) supports the expectation that supervised residents, fellows, or guest surgeons recorded procedures. In particular, the second sensitivity analysis yielded more reliable numbers, considering the anticipated numbers of Dutch MBS surgeons and surgeries performed per surgeon. As surgeons changing employers or residents transitioning to surgeons may receive new surgeon identifiers, the median number of surgeries performed per surgeon may still be slightly underestimated. Nevertheless, the similarity between the primary analysis and the sensitivity analyses suggests that combining surgeon identifiers for those surgeons would not significantly alter the results, as might be expected, given that, for example, residents are usually closely supervised during surgery by an experienced bariatric surgeon.

Where previous research has demonstrated outcome variation between hospitals^20,22,35–38^, the precise causes contributing to this variation often remain unclear. The present study highlights that, in the context of MBS, part of this variation is attributable to surgeon-level factors, particularly in relation to complications and reoperations. Still, a larger proportion is explained by hospital-level factors, most profoundly so for prolonged LOS. Factors that may underlie this association include the application of enhanced recovery protocols^39,40^, use of (local) anaesthetics, care coordination models (including interdisciplinary coordinating structures), and in-hospital paramedic support (for example dietetic support). When extending the perspective to other outcomes such as complications and weight loss, a former study suggested that hospitals may favour certain types of MBS based on institutional practices or policies, rather than patient factors or surgeon preferences^22^, which could explain part of the variance attributed to the hospital level. Other factors may include the preoperative workup (for example guidance by affiliated physiotherapists, dieticians, and psychologists), preoperative counselling, the expertise of nurses and ward physicians, hospital-wide protocols (for example perioperative anticoagulation management and discharge criteria), and the quality of postoperative care (for example remote consultations, accessibility and involvement of other specialists, and patient group meetings during follow-up). Efforts to improve MBS care may consider investigating these aspects in future studies.

The relatively low ICCs indicate that a substantial proportion of total outcome variability remains unexplained by the variables included in the model. Potential sources of this unexplained variance include factors such as patient behaviour, genetic predispositions, psychological conditions, and socioeconomic status^41–43^. Additionally, surgeon factors such as specialized training or completing a fellowship may also contribute. Although the ICCs at the surgeon level were low across all outcomes (for instance, 1.5% for reoperation), this does not necessarily imply negligible influence. It is possible that certain surgeon-level factors are indeed impactful but are not reflected in the variance because surgeons with these characteristics are evenly distributed across hospitals. As a result, their influence may be obscured in the multilevel model in the present study. Hence, further investigation into surgeon-level factors may be valuable for identifying opportunities to improve clinical outcomes. Nevertheless, some degree of variability may arise merely due to chance, as patients with identical clinical characteristics undergoing MBS by the same surgeon in the same hospital may still experience differing outcomes, for example with respect to complications. Therefore, a certain degree of variance is inherently unexplainable.

The present study has several limitations. First, data on some patient characteristics were not available, such as mobility limitations, prior venous thromboembolism, and smoking history, previously identified as risk factors for complications^3^, thereby potentially underestimating the impact of patient characteristics. Factors such as social environment, amount of disposable income, and psychological status could also play a role^43^. Nevertheless, given the magnitude of the differences in explained variance, it seems unlikely that adding these factors would considerably change the relative influence compared with surgeon- or hospital-level factors. Second, more granular data on surgeon and hospital characteristics were unavailable in the registry, limiting the authors’ understanding of how improvement may be achieved. Incorporating variables that reflect surgical training and, in particular, hospital protocols would enhance insight into this area. Nonetheless, the findings provide a useful direction for future research, especially with regard to exploring hospital-level factors. Finally, there may have been an underestimation of the ICCs, as they were calculated using π²/3 for the variance at the patient level. Had the true patient-level variance been known, it could have resulted in slightly more variance being explained^44^. However, it should be emphasized that the aim of the present study was not to discover the origin of all outcome variability. Although much of the variance remained unexplained, the primary aim was to assess the relative impact of the different levels compared with one another to decide whether feedback on performance is best directed at hospitals or surgeons.

A strength of the present study is that three levels were included in the multilevel analysis, whereas previous studies only studied two levels^31,32^. This enabled the assessment of the relative influence of both surgeon- and hospital-level factors in comparison with patient characteristics. Future research should investigate which factors within the hospital influence outcomes most, so that these care aspects can be targeted for further improvement. Keeping track of these care elements is therefore the most important first step.

In conclusion, hospital-level factors accounted for most of the variation in MBS outcomes, while surgeon-level factors contributed moderately, particularly to postoperative complications and reoperations. This indicates that hospital-level factors shared by all patients and surgeons, such as perioperative care, account for a greater proportion of the outcome variation than surgeon-level factors, such as surgical skill. It must be emphasized that Dutch MBS care is centralized, with dedicated bariatric surgeons performing MBS, so the results may be not be directly generalizable to other healthcare systems.

## Supplementary Material

znaf186_Supplementary_Data

## Data Availability

The data will not be made publicly available but can be made available upon appropriate request to the corresponding author.
