# Supplementary material for: Variation in outcomes after metabolic bariatric surgery: multilevel analysis to assess the contribution of patient, surgeon, and hospital factors
Source: Br J Surg. 2025 Oct 3;112(10):znaf186. doi: 10.1093/bjs/znaf186 (PMC12494228; doi:10.1093/bjs/znaf186)
Supplement: znaf186_Supplementary_Data [file znaf186_supplementary_data.docx]

Variation in outcomes after metabolic bariatric surgery: a multilevel analysis to assess the contribution of patient, surgeon, and hospital factors

Floris F.E. Bruinsma, MD ^a,b,†^; Simon W. Nienhuijs, MD PhD ^c^; Ronald S.L. Liem, MD ^d,e^; Jan Willem M. Greve, MD PhD ^a,f^; Perla J. Marang-van de Mheen, PhD ^g^; on behalf of the Dutch Audit for Treatment of Obesity Research Group

^a^ Department of Surgery, Maastricht University Medical Centre, NUTRIM School for Nutrition and Translational Research in Metabolism, Maastricht, The Netherlands

^b^ Scientific Bureau, Dutch Institute for Clinical Auditing, Leiden, The Netherlands

^c^ Department of Surgery, Catharina Hospital, Eindhoven, The Netherlands

^d^ Department of Surgery, Groene Hart Hospital, Gouda, The Netherlands

^e^ Nederlandse Obesitas Kliniek, The Hague and Gouda, The Netherlands

^f^ Weight Doctors Nederland, Quole, Waalre, The Netherlands

^g^ Safety & Security Science and Centre for Safety in Healthcare, Delft University of Technology, Delft, The Netherlands

^†^ Correspondence to: Floris F.E. Bruinsma, Department of Surgery, Maastricht University Medical Centre, P. Debyelaan 25, 6229 HX Maastricht, the Netherlands.

E-mail address: floris.bruinsma@maastrichtuniversity.nl

ORCID ID: 0000-0001-7975-3862

**Supplementary Materials - Index**

| **Supplementary Figures and Tables** |  |
| --- | --- |
| Table S1 | *pag. 2* |
|  |  |
|  |  |
|  |  |

**Supplementary Results**

**Supplementary file 1, Table S1: The proportion of explained variance attributable to each level according to the primary analysis and both sensitivity analyses.**

|  | $\boldsymbol{\propto}$V_expl_ | | | | | |
| --- | --- | --- | --- | --- | --- | --- |
|  | *Complication CD3+* | *Reoperation* | *Prolonged LOS* | *Readmission* | *Textbook Outcome* | *≥25% TWL* |
| **Patient** |  |  |  |  |  |  |
| A0 | 13.1% | 12.2% | 6.5% | 6.5% | 4.4% | 4.6% |
| A1 | 12.1% | 11.6% | 6.6% | 6.8% | 3.9% | 4.0% |
| A2 | 13.7% | 12.7% | 9.3% | 6.7% | 5.3% | 4.2% |
| **Surgeon** |  |  |  |  |  |  |
| A0 | 26.1% | 28.2% | 3.2% | 10.2% | 8.1% | 18.2% |
| A1 | 28.3% | 31.8% | 2.9% | 8.3% | 8.8% | 18.6% |
| A2 | 29.7% | 30.8% | 5.1% | 9.9% | 10.8% | 17.9% |
| **Hospital** |  |  |  |  |  |  |
| A0 | 60.8% | 59.6% | 90.3% | 83.3% | 87.5% | 77.2% |
| A1 | 59.7% | 56.6% | 90.5% | 84.9% | 87.3% | 77.3% |
| A2 | 56.6% | 56.5% | 85.6% | 83.4% | 83.9% | 77.8% |

$\boldsymbol{\propto}$V_expl_ = proportion of the explained variance, A0 = primary analysis, A1 = first sensitivity analysis, A2 = second sensitivity analysis, CD3+ = Clavien-Dindo grade 3 or higher, prolonged LOS = length of stay ≥ 3 days, Textbook outcome = composite outcome measure indicating no prolonged LOS and no readmissions or complications within first 30 days, %TWL = percentage total weight loss.
